# Three new species of *Inocybe* (Inocybaceae, Agaricales) from Southwestern China

**DOI:** 10.3897/mycokeys.133.195707

**Published:** 2026-06-05

**Authors:** Zi X. Yu, Zhu L. Yang

**Affiliations:** 1 State Key Laboratory of Phytochemistry and Natural Medicines, Kunming Institute of Botany, Chinese Academy of Sciences, Kunming 650201, Yunnan, China State Key Laboratory of Phytochemistry and Natural Medicines, Kunming Institute of Botany, Chinese Academy of Sciences Kunming China; 2 Yunnan Key Laboratory for Fungal Diversity and Green Development, Kunming Institute of Botany, Chinese Academy of Sciences, Kunming 650201, Yunnan, China Yunnan Key Laboratory for Fungal Diversity and Green Development, Kunming Institute of Botany, Chinese Academy of Sciences Kunming China; 3 College of Life Sciences, University of Chinese Academy of Sciences, Beijing 101408, China College of Life Sciences, University of Chinese Academy of Sciences Beijing China

**Keywords:** Basidiomycota, diversity, morphology, phylogeny, taxa, taxonomy

## Abstract

The genus *Inocybe* (Inocybaceae, Agaricales) is a globally diverse fungal lineage with significant ecological importance. In China, species belonging to *Inocybe* are widely distributed and exhibit exceptionally high diversity. However, the overall diversity of this genus in the country, particularly in the complex mountainous landscapes of southwestern China, remains severely underestimated. Here, based on morphological evidence and multi-locus (ITS, LSU, and *rpb2*) phylogenetic analyses, we describe three new species from the area, namely *Inocybe subtropicocinnamomea*, *I. biformis*, and *I. fulvobasalis*. Phylogenetically, *I. subtropicocinnamomea* is assigned to sect. *Inocybe* and occurs in subtropical broad-leaved or mixed forests. *Inocybe biformis* belongs to the smooth-spored temperate boreal clade (STBC) and is uniquely adapted to high-altitude subalpine coniferous habitats above 4000 m. *Inocybe fulvobasalis* is nested within the *I. geophylla* group and occurs in temperate broad-leaved and coniferous mixed forests. Detailed morphological descriptions, macroscopic photographs, line drawings, scanning electron micrographs of basidiospores, and taxonomic comparisons with allied taxa are provided for the novel species.

## Introduction

The genus *Inocybe* (Fr.) Fr., belonging to the family Inocybaceae (Agaricales), is one of the most species-rich ectomycorrhizal (ECM) fungal lineages worldwide (Matheny et al. 2006; Matheny 2009; Cripps et al. 2020; Corrales et al. 2022). By the end of March 2026, more than 1,000 species items of *Inocybe* have been recorded globally by the Fungal Names (FN) database (https://nmdc.cn/fungalnames/). Morphologically, *Inocybe* species are characterized by the predominantly small to medium-sized basidiomata; the dry pilei that are frequently fibrillose, squamulose, or rimose; the typically thick-walled cystidia; and basidiospores exhibiting a variety of shapes, ranging from smooth to nodulose or stellate. Additionally, many species emit a distinctive odor, such as a spermatic scent (Kuyper 1986; Matheny 2020, 2023; Matheny et al. 2020). Ecologically, members of *Inocybe* are widely distributed across climatic zones ranging from the cold temperate to the equatorial tropics. They form symbiotic associations with a diverse array of vascular plants, playing a crucial ecological role in nutrient cycling within forest ecosystems (Kuyper 1986; Singer 1986; Kobayashi 2002; Matheny et al. 2020; Bau 2022).

The name *Inocybe* was first introduced by Fries (1821) as a “tribe” (*Agaricus* tribus *Inocybe* Fr.) within the genus *Agaricus* L. It was later elevated to generic rank based on the ornamented (verrucose to angular) surfaces of the basidiospores observed in certain species (Fries 1863). Following this elevation, Schroeter (1889) and Fayod (1889) simultaneously segregated nodulose-spored species from smooth-spored ones, placing the former into the genera *Astrosporina* J. Schröt. and *Clypeus* (Britz.) Fayod, respectively. Rather than adopting this segregation, the majority of subsequent taxonomists adhered to Fries’s broader concept, maintaining *Inocybe* as a unified genus accommodating both smooth- and nodulose-spored taxa (Massee 1904; Heim 1931; Kühner 1933). Within this unified generic framework, Horak (1968) advocated for dividing the genus into two subgenera based on spore morphology: subgen. *Inocybe* (smooth-spored) and subgen. *Astrosporina* (ornamented-spored). Kuyper (1986) subsequently divided *Inocybe* into three subgenera—subgen. *Mallocybe* (basidiomata with yellow pigments), subgen. *Inosperma* (lacking pleurocystidia), and subgen. *Inocybe* (possessing pleurocystidia)—based on the presence or absence of pleurocystidia and yellow pigmentation.

In the 21^st^ century, the genus *Inocybe* s.l. was informally divided into five major clades: the *Inocybe* clade, the *Auritella* clade, the *Inosperma* clade, the *Mallocybe* clade, and the *Pseudosperma* clade (Matheny 2005, 2009; Alvarado et al. 2010; Matheny and Kudzma 2019). Matheny et al. (2020) then recognized these clades as genera. There are seven genera in Inocybaceae to date: *Inocybe* (Fr.) Fr., *Auritella* Matheny & Bougher, *Inosperma* (Kühner) Matheny & Esteve-Rav., *Mallocybe* (Kuyper) Matheny, Vizzini & Esteve-Rav., *Nothocybe* Matheny & K.P.D. Latha, *Pseudosperma* Matheny & Esteve-Rav., and *Tubariomyces* Esteve-Rav. & Matheny (Matheny et al. 2020).

In China, *Inocybe* species are widely distributed across various forest types from the northeast to the southwest, exhibiting exceptionally high species diversity (Li et al. 2021; Bau 2022; He et al. 2022). In recent years, more than 120 new species and new records have been successively reported from various regions of China, enriching our understanding of the species diversity and geographical distribution of *Inocybe* in East Asia (Fan and Bau 2010, 2014; Li et al. 2021; Bau 2022; He et al. 2022; Chen et al. 2024; Gao et al. 2024, 2025; Zhu et al. 2025; Li et al. 2026). Despite these advancements, the overall diversity of *Inocybe* in China remains severely underestimated, as indicated in other groups of macrofungi (Lu et al. 2025; Su et al. 2025; Wang et al. 2025). Comprehensive surveys of *Inocybe* resources are still lacking in many areas, particularly in southwestern China, leaving numerous cryptic and undescribed species yet to be discovered.

Here, three new species of *Inocybe* s. str. from southwestern China are reported based on an integration of morphological and phylogenetic evidence.

## Materials and methods

### Specimens and morphological observation

Specimens were collected in southwestern China. GPS coordinates, elevation, and habitat information were recorded in the field. Macroscopic features of fresh basidiomata were photographed using a digital camera. Color codes follow Kornerup and Wanscher (1978). Collected specimens were dried overnight in an electric oven at 45 °C, sealed in polyethylene bags, and deposited in the Cryptogamic Herbarium of the Kunming Institute of Botany, Chinese Academy of Sciences (KUN-HKAS). A small part of each collection was dehydrated using silica gel for molecular phylogenetic analysis.

Microscopic features of dried specimens were observed using a Zeiss AxioStar Plus microscope (Carl Zeiss, Germany). For observation, tissues were mounted in 5% KOH. Crystals at the apex of cystidia were additionally observed in water, and 1% Congo Red solution was used for staining when necessary. Basidiospore measurements were based on a minimum of 40 randomly selected mature basidiospores. The notation [n/m/p] indicates that measurements were made on n basidiospores from m basidiomata across p collections. Dimensions are presented in the format (a) b–*c*–d (e), where “a” and “e” represent extreme values, “b” to “d” represent the core range covering at least 90% of the measured values, and “*c*” represents the mean value. The length/width ratio (Q = spore length/width) was calculated, and the mean Q value and sample standard deviation are presented as Qm ± SD. For fine morphological observation of basidiospores, lamella fragments from dried specimens were rehydrated and then dehydrated through a graded ethanol series, critical-point dried, coated with a gold-palladium alloy, and observed under a Zeiss Sigma 300 scanning electron microscope (SEM) (Carl Zeiss).

### DNA extraction, PCR, and sequencing

For molecular phylogenetic studies, healthy, mold-free context tissues were selected from dried specimens. Total genomic DNA was extracted using the Ezup Column Fungi Genomic DNA Extraction Kit (Sangon Biotech, Shanghai, China) following the manufacturer’s instructions. Polymerase chain reaction (PCR) amplification was performed using the following primer pairs: ITS1F/ITS4 (White et al. 1990; Gardes and Bruns 1993) for the internal transcribed spacer (ITS) region, LR0R/LR5 (Vilgalys and Hester 1990) for the large subunit (LSU) of the ribosomal RNA gene, and bRPB2-6F/bRPB2-7R (Matheny 2005) for the second largest subunit of the RNA polymerase II (*rpb2*) gene. PCR was performed on a SimpliAmp™ Thermal Cycler. The amplification protocols followed previously published methods (Wang et al. 2025). Sanger sequencing was conducted by Sangon Biotech (Shanghai, China). Newly generated sequences were deposited in the NCBI GenBank database (https://www.ncbi.nlm.nih.gov/genbank/). Additional reference sequences used for phylogenetic analyses were retrieved from the same database.

### Sequence alignment and phylogenetic analysis

In total, 27 sequences were generated in this study (nine ITS, nine LSU, and nine *rpb2*). The newly generated sequences from the nine voucher specimens of the proposed new species were subjected to BLASTn similarity searches against the NCBI GenBank database to verify their identity and to retrieve homologous reference sequences. After excluding redundant sequences, the final dataset comprised 166 sequences. Four species from the genus *Mallocybe* were designated as the outgroup. Voucher specimen information, collection localities, and GenBank accession numbers for all sequences used in the phylogenetic analyses are provided in Table 1.

**Table 1.** Specimens used in phylogenetic analysis and GenBank accession numbers (Newly generated sequences are in bold).

Taxa	Voucher	Status	Location	GenBank accession number			Reference
				ITS	nrLSU	*rpb2*	
*Inocybe alberichiana*	DB12-9-19-16	Holotype	Austria	MW845855	MW845855	/	Bandini et al. (2021)
*I. alberichiana*	DB23-9-16-11		Germany	MW845856	MW845856	/	Bandini et al. (2021)
*I. amethystina*	L-0053531	Holotype	Netherlands	MW845932	/	/	Bandini et al. (2021)
*I. beatifica*	STU:SMNS-STU-F-0901261	Holotype	Germany	MW845857	/	/	Bandini et al. (2021)
*I. beatifica*	STU:SMNS-STU-F-0901471		Germany	MW845858	MW845858	/	Bandini et al. (2021)
*I. biformis*	KUN-HKAS 130371	Holotype	China	PZ280618	PZ280627	PZ284718	This study
*I. biformis*	KUN-HKAS 130400		China	PZ280619	PZ280628	PZ284719	This study
*I. biformis*	KUN-HKAS 99469		China	PZ280620	PZ280629	PZ284720	This study
*I. carolinensis*	TENN:071091	Holotype	USA	NR_165869	/	/	Matheny and Kudzma (2019)
*I. carolinensis*	TENN:067756		USA	KP636853	KP171055	KM555147	Matheny and Kudzma (2019)
*I. castorina*	DB21-10-15-2	Holotype	Germany	MN512319	MN512319	/	Bandini et al. (2020)
*I. costinitii*	MCVE 28974	Holotype	Croatia	KX686581	/	/	Bizio et al. (2016)
*I. derbschii*	KR-M-0005011	Holotype	Germany	MG012466	/	/	Bandini et al. (2019)
*I. derbschii*	DB3-6-18-Dondl		Germany	MW845868	/	/	Bandini et al. (2021)
*I. derbschii*	KR-M-0042367		Germany	MH366592	/	/	Bandini et al. (2019)
*I. dryadiana*	DB31-8-14-1	Holotype	Germany	MW845873	MW845873	/	Bandini et al. (2021)
*I. dryadiana*	DB2-8-14-11		Germany	MW845875	MW845873	/	Bandini et al. (2021)
*I. flocculosa*	DB1-10-12-16		Germany	MW856448	/	/	Bandini et al. (2021)
*I. flocculosa*	DB1-10-12-5		Germany	MW856449	MW856449	/	Bandini et al. (2021)
*I. fulvobasalis*	KUN-HKAS 154762	Holotype	China	PZ280621	PZ280630	PZ284721	This study
*I. fulvobasalis*	KUN-HKAS 154761		China	PZ280622	PZ280631	PZ284722	This study
*I. fulvobasalis*	KUN-HKAS 154763		China	PZ280623	PZ280632	PZ284724	This study
*I. fulvobasalis*	KUN-HKAS 130373		China	PZ280624	PZ280633	PZ284723	This study
*I. fuscicothurnata*	PBM3980		USA	MF487844	KY990485	MF416408	Matheny and Swenie (2018)
*I. fuscicothurnata*	AU9919	Isotype	Canada	KY923020	KY923039	/	Matheny and Swenie (2018)
*I. geophylla*	SMNS-STU-F-0901531	Epitype	Austria	MW845949	MW845949	/	Bandini et al. (2021)
*I. geophylla*	DB9-11-11-12		Germany	MW856426	/	/	Bandini et al. (2021)
*I. ghibliana*	STU:SMNS-STU-F-0901256	Holotype	Germany	MW845878	MW845878	/	Bandini et al. (2021)
*I. ghibliana*	STU:SMNS-STU-F-0901485		Germany	MW845880	MW845880	/	Bandini et al. (2021)
*I. griseovelata*	STU:SMNS-STU-F-0901568	Epitype	Germany	MW845942	MW845942	/	Bandini et al. (2021)
*I. griseovelata*	TENN:076184		USA	PQ658733	PQ600558	PQ625764	/
*I. grusiana*	STU:SMNS-STU-F-0901262	Holotype	Germany	MW845884	MW845884	/	Bandini et al. (2021)
*I. grusiana*	STU:SMNS-STU-F-0901487		Germany	MW845886	MW845886	/	Bandini et al. (2021)
*I. ionocephala*	TENN:062799	Holotype	USA	KY990551	KY990504	MF416422	Matheny and Swenie (2018)
*I. ionocephala*	TENN:062794		USA	KY990550	KY990503	MF416421	Matheny and Swenie (2018)
*I. juturnae*	STU:SMNS-STU-F-0901767	Holotype	Germany	OQ324780	OQ324780	/	Bandini et al. (2023)
*I. laurina*	DB23-10-16-6	Holotype	Germany	MN512325	MN512325	/	Bandini et al. (2020)
*I. laurina*	DB8-11-14-3		Germany	MW845947	MW845947	/	Bandini et al. (2020)
*I. lilacina*	TENN:062464		USA	KY990528	KY990484	MF416407	Matheny and Swenie (2018)
*I. lilacina*	NYS:f1711	Holotype	USA	MH024860	/	/	Matheny and Swenie (2018)
*I. maritimoides*	LVK22148		USA	PV090833	PV090833	PV107381	/
*I. maritimoides*	LVK14408		USA	PV090830	PV090830	PV107379	/
*I. nitidiuscula*	M 0229745	Epitype	Germany	KM873364	/	/	Marchetti et al. (2014)
*I. nivea*	HKAS 133520		China	PV715527	/	/	/
*I. nivea*	EL76-15		Norway	OK090776	OK090776	/	/
*I. oloris*	STU:SMNS-STU-F-0901681	Holotype	Germany	ON003437	ON003437	/	Bandini et al. (2022)
*I. pallidicremea*	PBM2039		USA	KY990553	AY380385	AY337388	Matheny and Swenie (2018)
*I. pallidicremea*	ACAD 11600	Isotype	Canada	KY923033	KY923042	/	Matheny and Swenie (2018)
*I. papilliformis*	CAL1374		India	KY440097	KY549127	/	Latha and Manimohan (2017)
*I. papilliformis*	TBGT:10480	Holotype	India	KP171131	KP170912	KM245988	Pradeep et al. (2016)
*I. parcecoacta*	ACAD11598	Isotype	Canada	KY923045	KY923040	/	/
*I. parcecoacta*	ACAD:10485		Canada	MH024852	MH024880	/	/
*I. plurabellae*	STU:SMNS-STU-F-0901260	Holotype	Germany	MW845901	MW845901	/	Bandini et al. (2021)
*I. plurabellae*	STU:SMNS-STU-F-0901501		Germany	MW845906	MW845906	/	Bandini et al. (2021)
*I. psammobrunnea*	MB89226	Holotype	France	MW845926	/	/	Bandini et al. (2021)
*I. relicina*	JV10258		Finland	AF325664	AY038324	AY333778	Peintner et al. (2001); Matheny (2005)
*I. relicina*	EL2-05			MN296111	MN296111	/	/
*I. semifulva*	WTU:ACAD11651	Holotype	Canada	HQ222006	HQ222007	/	/
*I. semifulva*	DB28-8-14-4		Germany	MW845916	MW845916	/	Bandini et al. (2021)
*I. sublilacina*	TENN:071464		Canada	KY990562	KY990520	MF416431	Matheny and Swenie (2018)
*I. sublilacina*	TENN:062542	Holotype	USA	/	KY990519	MF416430	Matheny and Swenie (2018)
*I. subtropicocinnamomea*	KUN-HKAS 145116	Holotype	China	PZ280616	PZ280625	PZ284716	This study
*I. subtropicocinnamomea*	KUN-HKAS 144698		China	PZ280617	PZ280626	PZ284717	This study
*I. tahquamenonensis*	WTU:43798	Holotype	USA	NR_164068	/	/	/
*I. tahquamenonensis*	MQ23-HRL2263		Canada	PP865776	/	/	/
*I. tigrina*	DB24-10-15-3	Epitype	Germany	MW845933	MW845933	/	Bandini et al. (2021)
*I. tigrina*	DB15-10-11-8		Germany	MW856437	/	/	Bandini et al. (2021)
*I. tubarioides*	PBM2550		USA	EU439453	AY732211	EU307855	/
*I. tubarioides*	HUH iNat#184973435		USA	PX652682	PX652885	/	/
*I. tubarioides*	HUH iNat#184734383		USA	PX652612	PX652815	/	/
*I. virgatula*	G:G00058741	Lectotype	France	MW845923	/	/	Bandini et al. (2021)
*I. virgatula*	KR KR-M-0038116		Germany	MW856438	/	/	Bandini et al. (2021)
*I. stellata*	CAL1369		India	KY440106	KY549136	KY553251	Latha and Manimohan (2017)
*I. stellata*	ECV3651 (SFSU)		Thailand	GQ893007	GQ892962	/	Horak et al. (2015)
*Mallocybe isabellina*	PERTH:08073287		Australia	KP171142	KP170921	KJ811587	Matheny and Bougher (2017)
*M. latispora*	TENN:063759		Finland	MN178505	MN178531	MN203522	Matheny et al. (2023)
*M. terrigena*	EL 117-04		Sweden	AM882864	AY380401	AY333309	Ryberg et al. (2008); Matheny (2005)
*M. tomentosula*	PBM 4138		USA	MG773814	MK421969	MH577506	/

Maximum likelihood (ML) phylogenetic analysis was performed using IQ-TREE v3.0.1 (Nguyen et al. 2015). The concatenated dataset was partitioned into three regions (ITS, LSU, and *rpb2*) utilizing an edge-linked partition model (-spp option). The best-fit evolutionary models for each partition were automatically evaluated and selected by the implemented ModelFinder tool (-m MFP option), which yielded TVM+F+I+G4 for ITS, TIM2+F+I+G4 for LSU, and TPM2+I+G4 for *rpb2*. Branch support for the ML tree was assessed using 1,000 ultrafast bootstrap (UFB) replicates along with 1,000 SH-like approximate likelihood ratio test (SH-aLRT) replicates. Bayesian inference (BI) was performed using MrBayes v3.2.7a (Ronquist et al. 2012), based on the optimal models selected by IQ-TREE. The Markov Chain Monte Carlo (MCMC) algorithm was executed with two independent runs, each consisting of four chains (one cold and three heated). The MCMC analysis was run for 10,000,000 generations, with trees sampled every 1,000 generations, and substitution rates were allowed to vary independently across partitions. Run convergence was confirmed when the average standard deviation of split frequencies (ASDSF) fell below 0.01 and the effective sample sizes (ESS) for all parameters were greater than 200. The initial 25% of the sampled trees from each run were discarded as the burn-in phase. The remaining trees were used to compute a 50% majority-rule consensus tree and to calculate the Bayesian posterior probabilities (PP). The resulting phylogenies were visualized in FigTree v1.4.4 (Rambaut 2018) and iTOL v7 (Letunic and Bork 2021), and subsequently edited for publication using Adobe Illustrator 2022.

## Results

### Phylogenetic results

**Figure 1.**
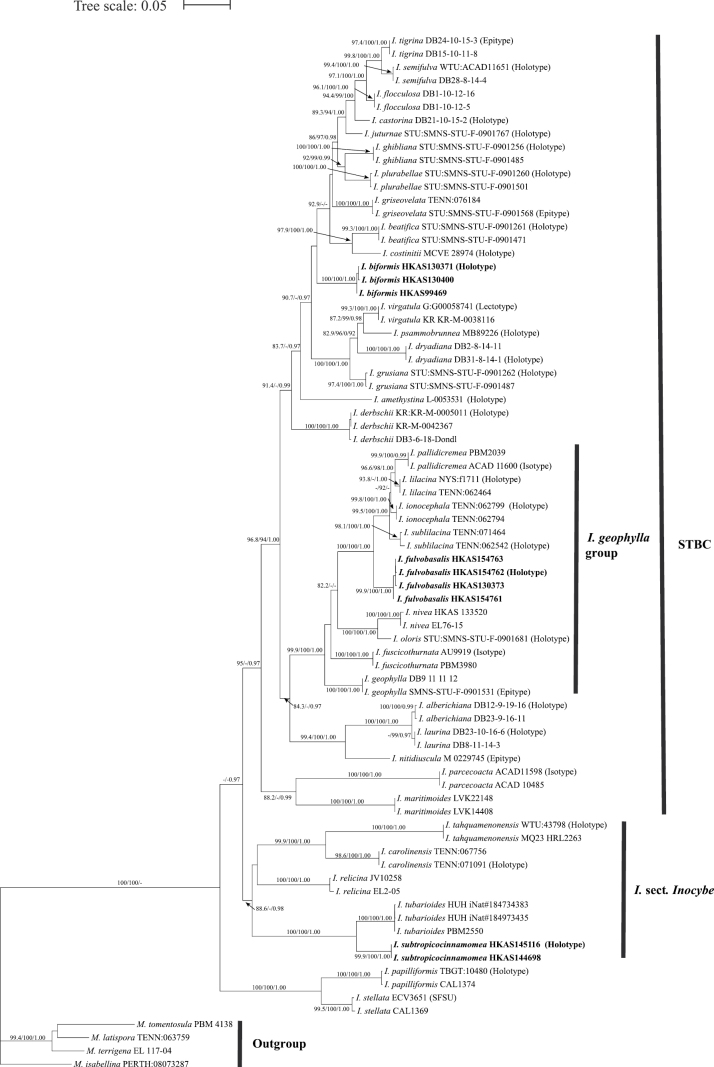
Maximum-likelihood phylogenetic tree inferred from a combined ITS, LSU and *rpb2* dataset, with *Mallocybe* species as the outgroup. SH-aLRT support values ≥ 80%, UFB values ≥ 90%, and BPP values ≥ 0.90 are shown above the nodes as SH-aLRT/UFB/BPP. Sequences of the newly described species are shown in bold.

The combined multi-locus sequence dataset (ITS, LSU, and *rpb2*) consisted of 78 terminal taxa. The total alignment length was 2502 base pairs (bp), comprising 876 bp of the ITS region, 915 bp of the LSU region, and 711 bp of the *rpb2* region. Within this alignment, 823 sites were identified as parsimony-informative. The topologies recovered from the ML and BI analyses were highly congruent. Accordingly, only the optimal ML tree is shown (Fig. 1), with SH-aLRT support, ultrafast bootstrap (UFB) values, and Bayesian posterior probabilities (BPP) annotated at corresponding nodes (SH-aLRT/UFB/BPP). Statistical support is annotated at the nodes only when SH-aLRT values are ≥ 80%, UFB values are ≥ 90%, and BPP are ≥ 0.90.

### Taxonomy

#### Inocybe subtropicocinnamomea

Taxon classificationFungiAgaricalesInocybaceae

Zhu L. Yang & Zi X. Yu sp. nov.

A3E91353-66D9-5BF9-B18F-142D820C5917

863690

Fig. 2

##### Etymology.

The epithet “*subtropicocinnamomea*” refers to the cinnamon-brown coloration of the pileus and the subtropical habitat of this species.

**Figure 2.**
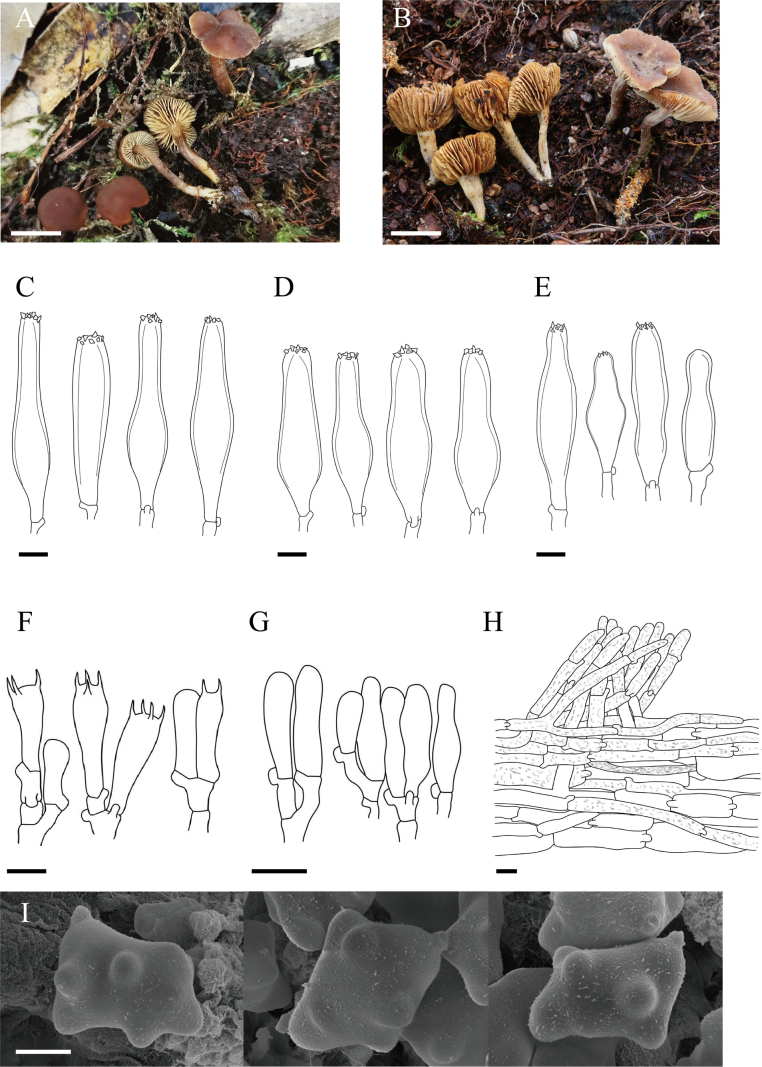
*Inocybe subtropicocinnamomea*KUN-HKAS 145116 holotype (**A, C–I**), KUN-HKAS 144698 (**B**). **A, B**. Basidiomata; **C**. Pleurocystidia; **D**. Cheilocystidia; **E**. Caulocystidia; **F**. Basidia; **G**. Cheiloparacystidia; **H**. Pileipellis; **I**. Basidiospores. Scale bars: 1 cm (**A, B**); 10 μm (**C–G**); 5 μm (**H**); 2 μm (**I**).

##### Diagnosis.

*Inocybe subtropicocinnamomea* is close and similar to *I. tubarioides* G.F. Atk., but differs in having a subumbonate, darker-colored pileus with less obvious discoloration when fading and relatively more slender and thinner-walled pleurocystidia.

##### Holotype.

China • Yunnan, Chuxiong Yi Autonomous Prefecture, Nanhua County, Ailaoshan National Nature Reserve, Dazhongshan Protection Station, in a subtropical broad-leaved forest with trees of *Lithocarpus xylocarpus*, 24°51'52"N, 100°48'14"E, alt. 2583 m, 13 August 2024, Xiang-Hua Wang 11865 (KUN-HKAS 145116. GenBank no.: ITS: PZ280616; LSU: PZ280625; *rpb2*: PZ284716).

##### Description.

***Basidiomata*** small. ***Pileus*** 6–16 mm in diameter, convex with a subumbonate center when mature, applanate with a depressed center when old; surface dry, cinnamon brown (7E4–7E6) or dark brown (7F2–7F5), dark brown (7F2–7F5) towards the center, pale brown (5D7, 5E7) towards the margin when old, covered with furfuraceous or appressed-fibrillose squamules; margin initially inflexed, later expanding to straight, becoming slightly reflexed and undulate with age, pale yellow (1A2, 1A3); context of pileus pale ochraceous (6C6, 6D7). ***Lamellae*** adnate to emarginate, moderately crowded, 2–3 mm broad, with 1–3 tiers of lamellulae, subventricose, pale yellow (1A2, 1A3) when young, orangish yellow (5A4–5A6) or yellow-brown (5C6–5C8) with age; edge often uneven, fimbriate, concolorous with or paler than the lamellae surface. ***Stipe*** 11–18 × 1.5–2.5 mm, cylindrical, occasionally tapering downwards when old, with the apex of the stipe slightly swollen up to 4 mm wide; surface pale brown (5B4, 5C4) at the apex, deepening to dark brown (6E3–6E5) downward, pale yellow (2A2–2A4) near the base, but overall becoming orangish brown (5A6, 5C6) to brown (5D6, 5E6), intermixed with dirty white (3A1, 3B1) when old; the apical part of stipe pruinose; context of stipe whitish.

***Basidiospores*** [80/2/2] 6.0–7.0–8.0 × 5.0–5.5–6.0 μm, Q = 1.10–1.22–1.40, Qm ± SD = 1.22 ± 0.12, slightly thick-walled, angular to polygonal with 5–7 distinct nodules up to 1–2 µm long, pale yellow to yellow in 5% KOH, typically with a prominent central lipid droplet; germ pore absent; apiculus small and inconspicuous. ***Basidia*** 14–30 × 5–8 µm, clavate to narrowly clavate, hyaline in 5% KOH, mostly 4-spored, sometimes 2-spored; sterigmata up to 4 µm long. ***Pleurocystidia*** 50–86 × 8–17 µm, narrowly lageniform to narrowly utriform, occasionally fusiform, slightly thick-walled (ca. 0.5 μm thick), hyaline; apices obtuse and crystalliferous. ***Cheilocystidia*** 43–74 × 9–18 µm, similar to pleurocystidia but somewhat shorter and with a relatively thicker neck, yellow or hyaline in 5% KOH. ***Cheiloparacystidia*** 9–26 × 4–8 µm, clavate to narrowly utriform, occasionally lecythiform, thin-walled, smooth, hyaline, mixed with cheilocystidia. ***Caulocystidia*** 38–65 × 9–14 µm, ellipsoidal to fusiform, slightly thick-walled (ca. 0.5–1 µm thick), occasionally septate, hyaline or yellow in 5% KOH; apices obtuse. ***Cauloparacystidia*** 12–18 × 8–14 µm, ellipsoidal, clavate to globose, in clusters, thin-walled, hyaline. ***Pileipellis*** a cutis composed of cylindrical to inflated, thin-walled, hyaline to pale yellow hyphae 3–15 μm wide, often with secondary unclamped septa; squamules on the pileipellis surface composed of frequently septate hyphae in fascicle, 3–7 μm wide, often with yellow cytoplasmic pigment. ***Pileal trama*** subregularly arranged, composed of cylindrical, thin-walled, hyaline to pale yellow inflated hyphae 9–15 μm wide. ***Hymenophoral trama*** 60–120 µm thick, subregularly arranged, colorless, composed of thin-walled, smooth, frequently septate, inflated hyphae 5–25 µm wide. ***Stipitipellis*** regularly arranged, composed of cylindrical, frequently septate, hyaline hyphae 3–7 μm wide. ***Clamp connections*** common in all tissues.

##### Habitat.

Solitary or gregarious on soil in subtropical broad-leaved forests or broad-leaved and coniferous mixed forests dominated by trees of *Quercus* and *Pinus*; summer.

##### Distribution.

Currently known only from southwestern China.

##### Additional specimen examined.

China • Yunnan, Chuxiong Yi Autonomous Prefecture, Nanhua County, Ailaoshan National Nature Reserve, Dayangqi Protection Station, in a subtropical broad-leaved and coniferous mixed forest with trees of *Pinus armandii*, *P. yunnanensis* and *Quercus variabilis*, 24°54'52"N, 100°46'06"E, alt. 2459 m, 10 August 2024, Xiang-Hua Wang 11609 (KUN-HKAS 144698).

##### Notes.

*Inocybe subtropicocinnamomea* is characterized by its cinnamon brown to dark brown, subumbonate pileus with furfuraceous squamules.

Phylogenetically, this species belongs to *Inocybe* sect. *Inocybe* (Singer 1986; Matheny and Moreau 2009; Matheny and Kudzma 2019). In the sect. *Inocybe*, *I. subtropicocinnamomea* forms a well-supported sister clade with the North American species *I. tubarioides*. However, *I. tubarioides* differs from *I. subtropicocinnamomea* by its convex to plane pileus without a distinct umbo, which fades to a pale color (Matheny and Moreau 2009). Within the same section, *I. subtropicocinnamomea* can be readily differentiated macroscopically from *I. carolinensis* Matheny & Kudzma, *I. tahquamenonensis* D.E. Stuntz, and *I. relicina* (Fr.) Fr. solely by the color of its pileus and lamellae. Specifically, while *I. subtropicocinnamomea* has a cinnamon brown to dark brown pileus and pale yellow to orangish yellow lamellae, *I. carolinensis* possesses a reddish brown to dark red pileus and pinkish gray to dark red lamellae, *I. tahquamenonensis* exhibits a dark fuscous purple pileus and vinaceous lamellae, and *I. relicina* is characterized by a dark umbrinous pileus with lamellae that are yellow only in youth (Quélet 1888; Stuntz 1954; Moser 1978; Matheny and Kropp 2001; Matheny and Kudzma 2019).

#### Inocybe biformis

Taxon classificationFungiAgaricalesInocybaceae

Zhu L. Yang & Zi X. Yu sp. nov.

D79C0847-718F-50C8-8325-C4485A200CCD

863691

Fig. 3

##### Etymology.

The epithet “*biformis*” refers to the squamules that differ in shape, and arrangement across the pileus.

**Figure 3.**
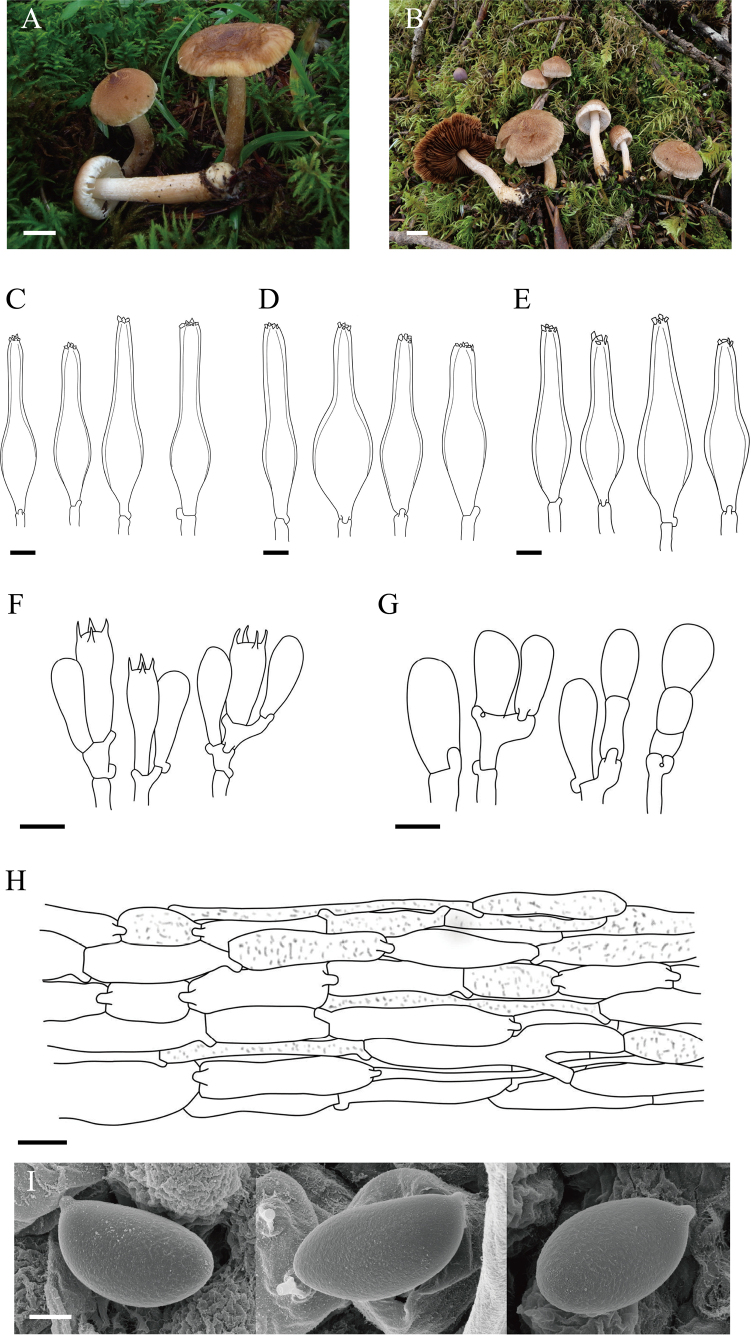
*Inocybe biformis*KUN-HKAS 130371 holotype (**B, C–I**), KUN-HKAS 99469 (**A**). **A, B**. Basidiomata; **C**. Pleurocystidia; **D**. Cheilocystidia; **E**. Caulocystidia; **F**. Basidia; **G**. Cheiloparacystidia; **H**. Pileipellis; **I**. Basidiospores. Scale bars: 1 cm (**A, B**); 10 μm (**C–H**); 2 μm (**I**).

##### Diagnosis.

*Inocybe biformis* looks like *I. beatifica* Bandini & B.Oertel, but distinguished from the latter by its rougher pileus with imbricate squamules at the center, distinctly darker lamellae and restriction to high elevations above 4000 m.

##### Holotype.

China • Xizang, Linzhi, Zayu County, Chawalong Township, Jiaying Village, in a temperate subalpine coniferous forest with trees of *Abies* and *Rhododendron*, 28°30'59"N, 98°31'29"E, alt. 4068 m, 11 August 2022, Xiang-Hua Wang 10446 (KUN-HKAS 130371. GenBank no.: ITS: PZ280618; LSU: PZ280627; *rpb2*: PZ284718).

##### Description.

***Basidiomata*** medium-sized to large. ***Pileus*** 20–50 mm in diameter, hemispherical when young, gradually becoming applanate with age; surface dry, yellowish brown (5B6, 5C6) to brown (5C8, 5D8), covered with imbricate squamules at the disc, transitioning to fibrils towards the margin; margin involute when young, with white (1A1) membranous velipellis remnants, becoming straight with age, often irregularly rimose when old, white (1A1) to yellowish brown (4B5–4B8); context of pileus dull whitish (5B2) to slightly yellowish brown (5B3–5B5), odor spermatic. ***Lamellae*** adnate, crowded, 3–5 mm broad, alternately distributed with 1–3 tiers of lamellulae, subventricose, white (1A1) when young, pale brown (2B4–2B6) to dark brown (5D8, 5E8) with age; edge often uneven, fimbriate, concolorous with or paler than the lamellae surface. ***Stipe*** 35–60 × 4–7 mm, cylindrical, yellowish brown (5C5–5C7), with a subbulbous base up to 9 mm wide; apex pruinose with white (1A1) granules, becoming fibrillose to squamulose below, yet retaining sparse residual pruina; context of stipe stuffed, ochraceous (6C8, 6E8).

***Basidiospores*** [100/3/3] (8.5–)9.0–10.0–10.5(–11.0) × 5.0–6.0–6.5 μm, Q = 1.54–1.67–1.90, Qm ± SD = 1.67 ± 0.12, ellipsoid to slightly amygdaliform, pale yellowish brown in KOH, slightly thick-walled, smooth, typically with a prominent central lipid droplet; germ pore absent; apiculus small but distinct. ***Basidia*** 16–28 × 7–10 µm, mostly clavate to broadly clavate, occasionally oblong or fusiform, hyaline or pale yellowish brown in 5% KOH, mostly 4-spored, sometimes 2-spored; sterigmata up to 4 µm long. ***Pleurocystidia*** 60–87 × 12–22 µm, mostly narrowly lageniform with a basal pedicel, occasionally broadly conical, thick-walled (ca. 1.5–3 μm thick), pale yellowish brown or hyaline in 5% KOH; apices obtuse and crystalliferous. ***Cheilocystidia*** 55–80 × 14–19 µm, similar to pleurocystidia, but sometimes with a more inflated ventricose portion, thick-walled, walls colorless to yellowish. ***Cheiloparacystidia*** 10–25 × 7–15 µm, broadly clavate, elliptical or subglobose, thin-walled, smooth, hyaline, mixed with cheilocystidia. ***Caulocystidia*** 55–85 × 16–22 µm, similar to pleurocystidia but slightly broader overall. ***Cauloparacystidia*** 9–35 × 4–15 µm, highly variable in shape, globose, clavate or elliptical, thin-walled, hyaline, abundant. ***Pileipellis*** a cutis, composed of cylindrical to pyriform, thin-walled, hyaline to pale yellow hyphae 3–25 μm wide. ***Pileal trama*** subregularly arranged, composed of thin-walled, hyaline hyphae 12–30 μm wide. ***Hymenophoral trama*** 120–260 µm thick, subregularly arranged, composed of cylindrical, clavate, or slightly inflated to subglobose, rarely pyriform, thin-walled hyphae 5–25 μm wide, hyaline, or occasionally pale yellow. ***Stipitipellis*** regularly arranged, composed of cylindrical, hyaline to pale yellow hyphae 3–12 μm wide. ***Clamp connections*** common in all tissues.

##### Habitat.

Gregarious on soil in coniferous forests associated with *Abies* and *Rhododendron*; summer.

##### Distribution.

Currently known only from southwestern China.

##### Additional specimens examined.

China • Xizang, Linzhi, Zayu County, Chawalong Township, Jiaying Village, in a coniferous forest with trees of *Abies* and *Rhododendron*, 28°30'58"N, 98°31'29"E, alt. 4075 m, 11 August 2022, Xiang-Hua Wang 10475 (KUN-HKAS 130400); China • Sichuan, Ganzi Autonomous Prefecture, Kangding City, Yala Township, Mugecuo Nature Reserve, in a coniferous forest with trees of *Abies*, 30°10'13"N, 101°52'20"E, alt. 4050 m, 9 September 2016, Bang Feng 115 (KUN-HKAS 99469).

##### Notes.

*Inocybe biformis* is characterized by a yellowish brown to brown pileus with imbricate squamules at the disc, transitioning to fibrils towards the margin, and a pruinose stipe.

Phylogenetically, *I. biformis* belongs to the smooth-spored temperate boreal clade (STBC) (Matheny 2009; Matheny et al. 2020). BLAST searches of the ITS sequence of the *I. biformis* holotype indicate a close match to *I. juturnae*, with a sequence similarity of 90.9% to the holotype of that species. However, *I. juturnae* differs from *I. biformis* by its warm yellow and smooth to minutely tomentose pileus (Bandini et al. 2023). Morphologically, *I. biformis* could be mistaken for *I. beatifica*, but the latter differs by having a relatively smoother pileus surface, paler lamellae at maturity, and uniformly white context in both the pileus and stipe (Bandini et al. 2021). *Inocybe costinitii* Bizio, Ferisin & Dovana features more distant lamellae and almost neckless hymenial cystidia, contrasting with the narrowly lageniform cystidia of *I. biformis* (Bizio et al. 2016).

Moreover, *I. biformis* shares a squamulose or felty pileus texture with *I. plurabellae* Bandini, B. Oertel & U. Eberh., *I. flocculosa* Sacc., and *I. tigrina* R. Heim. However, *I. plurabellae* exhibits highly variable pileus coloration and a finely roughened pileus surface (Bandini et al. 2021); *I. flocculosa* has a more uniform, less variable pileus color, a (sub) squamulose pileus texture, and a distinctly paler stipe at maturity (Kuyper 1986; Stangl 1989; Bandini et al. 2021); *I. tigrina* typically exhibits a bicolored, felty to sublanose pileus with diffracted fibers as well as consistently narrower basidiospores (Heim 1931; Ferrari 2006; Ludwig 2017; Bandini et al. 2021). Ecologically, *I. biformis* is uniquely adapted to high-altitude environments (over 4000 m), whereas the other species are predominantly distributed in Europe and often associate with different hosts at lower altitudes.

#### Inocybe fulvobasalis

Taxon classificationFungiAgaricalesInocybaceae

Zhu L. Yang & Zi X. Yu sp. nov.

3368DE2A-371E-5FFD-8911-312044EA378A

863672

Fig. 4

##### Etymology.

The epithet “*fulvobasalis*” refers to the characteristic persistently yellow-brown base of the stipe throughout the ontogeny of this species.

**Figure 4.**
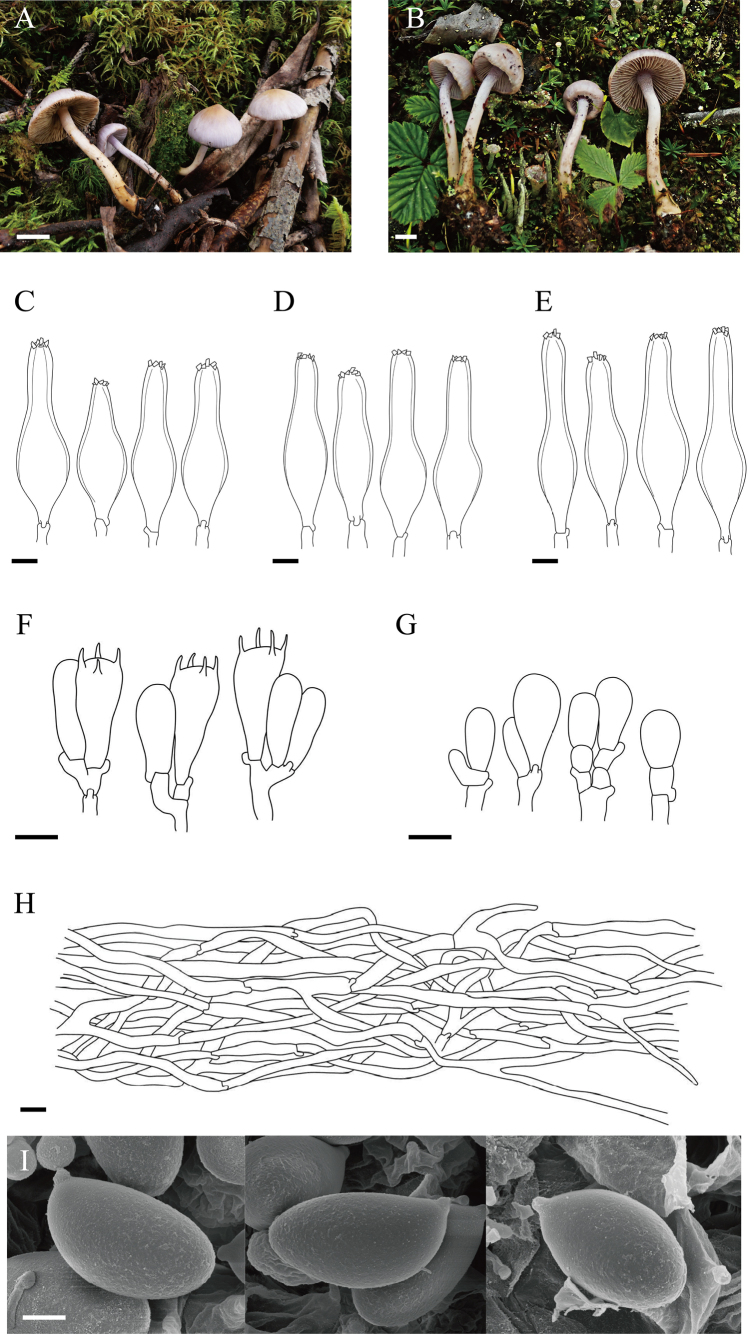
*Inocybe fulvobasalis*KUN-HKAS 154762 holotype (**B, C–I**), KUN-HKAS 130373 (**A**). **A, B**. Basidiomata; **C**. Pleurocystidia; **D**. Cheilocystidia; **E**. Caulocystidia; **F**. Basidia; **G**. Cheiloparacystidia; **H**. Pileipellis; **I**. Basidiospores. Scale bars: 1 cm (**A, B**); 10 μm (**C–H**); 2 μm (**I**).

##### Diagnosis.

*Inocybe fulvobasalis* can be distinguished from other species in the *I. lilacina* subgroup by its grayish lilac pileus, which is hemispherical to subumbonate when young, as well as its stipe with a persistently yellow-brown base throughout ontogeny.

##### Holotype.

China • Yunnan, Diqing Zang Autonomous Prefecture, Shangri-La City, Potatso National Park, in a temperate broad-leaved and coniferous mixed forest with trees of *Picea*, *Abies* and *Quercus acutissima*, 27°52'48"N, 99°57'00"E, alt. 3613 m, 11 September 2023, Wei-Chao Feng 0064 (KUN-HKAS 154762. GenBank no.: ITS: PZ280621; LSU: PZ280630; *rpb2*: PZ284721).

##### Description.

***Basidiomata*** small to medium-sized. ***Pileus*** 10–40 mm in diameter, hemispherical to subumbonate when young, gradually expanding to convex or plano-convex with age, often with an indistinct central umbo; surface hygrophanous, pale lilac (15A2, 15A3) to grayish lilac (16B2, 16B3) throughout when young, with the umbo often tinged with pale brownish (4B5–4B8) buff, the disc and umbo fade to yellowish (3A3–3A6) with age; margin initially incurved, becoming depressed to straight with age, often retaining faint lilac (15A2, 15A3) tones, occasionally with an orange-brown tinge (6A3–6A5), silky-fibrillose; context of pileus white (1A1) to pale whitish gray (1B1), odor strongly spermatic. ***Lamellae*** emarginate to sinuate, crowded, 3–5 mm broad, alternately distributed with 1–3 tiers of lamellulae, subventricose, pale grayish lilac (15A2, 15B2) when young, becoming pale yellowish (4A2–4A4) when old; edges indistinctly pallid-fimbriate. ***Stipe*** 37–87 × 2–5 mm, cylindrical, pale grayish lilac (15A2, 15B2) when young, the apex bearing the most intense lilac tones, occasionally becoming pale yellow (1A2–1A5) overall with age, the base consistently tinged with stable yellowish brown (3A5, 3B5) to light brownish (4A7, 4B7) buff; with upper 1/8–1/6 distinctly pruinose, faint longitudinal fibrillose striations present towards the base; cortina fugacious, occasionally leaving fugacious, ephemeral annular remnants on the upper stipe.

***Basidiospores*** [100/4/4] (9.0)9.5–10.0–11.0(12.0) × (5.0)5.5–6.0–7.0(7.2) μm, Q = (1.5)1.57–1.67–1.83(2.2), Qm ± SD = 1.67 ± 0.13, ellipsoid to slightly amygdaliform, yellowish brown in KOH, slightly thick-walled, smooth, typically with a prominent central lipid droplet; germ pore absent; apiculus small but distinct. ***Basidia*** 15–32 × 6–13 µm, narrowly clavate to clavate, hyaline in 5% KOH; mostly 4-spored, sometimes 2-spored; sterigmata up to 5 µm long. ***Pleurocystidia*** 52–75 × 16–22 µm, narrowly utriform or fusiform with a basal pedicel, occasionally ovoid, thick-walled (ca. 0.5–2 μm thick), pale yellowish green or hyaline in 5% KOH; apices obtuse and crystalliferous. ***Cheilocystidia*** 55–75 × 13–20 µm, similar to pleurocystidia, but sometimes with a more slender and elongated neck. ***Cheiloparacystidia*** 8–27 × 5–20 µm, clavate, elliptical or subglobose, thin-walled, smooth, hyaline, mixed with cheilocystidia. ***Caulocystidia*** 53–95 × 10–19 µm, similar to hymenial cystidia, mostly narrowly lageniform. ***Cauloparacystidia*** 13–35 × 9–15 µm, clavate, elliptical or subglobose, thin-walled to slightly thickened, hyaline, abundant. ***Pileipellis*** a cutis, composed of slender and cylindrical, thin-walled, hyaline hyphae 3–8 μm wide. ***Pileal trama*** subregularly arranged, composed of thin-walled hyphae 7–25 μm wide. ***Hymenophoral trama*** regularly to subregularly arranged, composed of cylindrical to clavate, thin-walled, smooth, hyaline hyphae 3–15 μm wide. ***Stipitipellis*** regularly arranged, composed of cylindrical, hyaline to pale yellow hyphae 5–16 μm wide. ***Clamp connections*** common in all tissues.

##### Habitat.

Gregarious on soil in temperate broad-leaved and coniferous mixed forests with trees of *Picea*, *Abies* and *Quercus acutissima*, or in coniferous forests with trees of *Abies* and *Rhododendron*.

##### Distribution.

Currently known only from southwestern China.

##### Additional specimens examined.

China • Yunnan, Diqing Zang Autonomous Prefecture, Shangri-La City, Potatso National Park, in a temperate broad-leaved and coniferous mixed forest with trees of *Picea*, *Abies* and *Quercus acutissima*, 27°52'48"N, 99°57'00"E, alt. 3521 m, 11 September 2023, Wei-Chao Feng 0045 (KUN-HKAS 154761); China • Yunnan, Diqing Zang Autonomous Prefecture, Shangri-La City, near Longmu Middle Bridge, East Ring Road, in a temperate broad-leaved and coniferous mixed forest with trees of *Picea*, 27°42'18"N, 100°00'39"E, alt. 3474 m, 13 September 2023, Ze-Wei Liu 291 (KUN-HKAS 154763); China • Xizang, Linzhi, Zayu County, Chawalong Township, Jiaying Village, in a coniferous forest with trees of *Abies* and *Rhododendron*, 28°30'59"N, 98°31'29"E, alt. 4027 m, 11 August 2022, Xiang-Hua Wang 10448 (KUN-HKAS 130373).

##### Notes.

*Inocybe fulvobasalis* is characterized by a grayish lilac pileus, with the umbo often tinged with pale brownish buff, and a grayish lilac stipe with a persistently yellow-brown base.

Phylogenetically, *I. fulvobasalis* is located in the *I. geophylla* (Fr.) P. Kumm. group and forms a sister clade to the *I. lilacina* subgroup, which consists of *I. pallidicremea* Grund & D.E. Stuntz, *I. ionocephala* Matheny, *I. lilacina* (Peck) Kauffman and *I. sublilacina* Matheny & A. Voitk (Matheny 2005; Ryberg et al. 2010; Matheny and Swenie 2018). Morphologically, *I. fulvobasalis* is easily confused with *I. pallidicremea* and *I. ionocephala*. However, *I. pallidicremea* differs in often being obtusely conical when young, having a deeper purple coloration, a more prominent umbo at maturity, and slightly smaller basidiospores (9.0 × 5.3 μm vs. 10.0 × 6.0 μm) (Grund and Stuntz 1977; Lincoff 1981; Matheny and Swenie 2018); *I. ionocephala* is separated by its white stipe throughout the entire ontogeny (Matheny and Swenie 2018). *Inocybe lilacina* can be distinguished by its brighter, more vivid overall purple coloration, and a deeply purple pileus disc that is significantly more persistent through development (Peck 1874; Kauffman 1918, 1924; Matheny and Swenie 2018). *Inocybe sublilacina* differs in its conical pileus in young basidiomata, and the stipe base that fades to white at maturity (Matheny and Swenie 2018).

## Discussion

### Phylogenetics of the three new species within *Inocybe*

In this study, three new species (*I. subtropicocinnamomea, I. biformis, I. fulvobasalis*) of *Inocybe* are described. Our combined ITS-LSU-*rpb2* phylogenetic analysis reveals that all three taxa are resolved as distinct lineages and separate from previously described species within the genus. *Inocybe subtropicocinnamomea* is assigned to sect. *Inocybe* (Kuyper 1986; Singer 1986; Matheny 2009; Matheny and Kudzma 2019). However, statistical support for the phylogenetic placement of its corresponding lineage in our multi-locus phylogeny remains moderate (88.6/-/0.98), suggesting that the deep-node evolutionary relationships within this section warrant further investigation. Within this section, *I. subtropicocinnamomea* forms a strongly supported sister clade (100/100/1.00) with the North American species *I. tubarioides*. *Inocybe fulvobasalis* belongs to the *I. geophylla* group, forming a strongly supported (100/100/1.00) independent branch that is sister to the *I. lilacina* subgroup (Matheny 2005; Ryberg et al. 2010; Matheny and Swenie 2018). Meanwhile, *I. biformis* is nested within the smooth-spored temperate boreal clade (STBC). The exact phylogenetic position of *I. biformis* within the genus *Inocybe* remains to be fully clarified and will require broader taxon sampling in future studies.

### Ecological distribution of the three new species in southwestern China

Ecologically, the three new species occupy different ecological niches and climatic zones. Interestingly, this ecological divergence locally correlates with their basidiospore morphology. The nodulose-spored *I. subtropicocinnamomea* is found in subtropical broad-leaved or mixed forests, forming associations with plants of *Quercus* and *Pinus* at intermediate elevations around 2400–2600 m. Conversely, the two smooth-spored species, *I. biformis* and *I. fulvobasalis*, are adapted to temperate subalpine environments. *Inocybe fulvobasalis* typically occurs in temperate broad-leaved and coniferous mixed forests with *Picea*, *Abies*, and *Quercus* at elevations ranging from 3400 to over 4000 m. Notably, *I. biformis* appears uniquely adapted to harsh high-altitude environments, exclusively found in subalpine coniferous forests associated with *Abies* and *Rhododendron* at elevations exceeding 4000 m, whereas its morphologically similar counterparts are predominantly distributed in Europe at lower altitudes (Bandini et al. 2021).

While basidiospore ornamentation is not a universal predictor of elevational preference—as nodulose-spored *Inocybe* species are well-documented in extreme alpine zones elsewhere, such as the Rocky Mountains (Cripps et al. 2020), and morphological traits often exhibit complex evolutionary histories (Ryberg et al. 2010), this localized pattern in southwestern China is a noteworthy observation. It suggests that the highly heterogeneous mountainous landscapes and isolated microclimates of this region may have driven distinct ecological partitioning and host co-evolution for these specific smooth- and nodulose-spored lineages.

## Supplementary Material

XML Treatment for Inocybe subtropicocinnamomea

XML Treatment for Inocybe biformis

XML Treatment for Inocybe fulvobasalis

## Additional information

### Artificial Intelligence (AI) use

The authors accept full responsibility for the content of the manuscript, including the disclosure of any use of AI.

No AI tools were used in the preparation of this manuscript.

### Author ORCIDs

Z.X. Yu https://orcid.org/0009-0008-4020-5701

Z.L. Yang https://orcid.org/0000-0001-9745-8453

### Data availability

Data underlying the results of this study are provided within the manuscript. All newly obtained nucleotide sequences have been submitted to the NCBI GenBank database.
